# Focal and diffuse myocardial fibrosis both contribute to regional hypoperfusion assessed by post-processing quantitative-perfusion MRI techniques

**DOI:** 10.3389/fcvm.2023.1260156

**Published:** 2023-09-19

**Authors:** Jeremy Weiner, Corinna Heinisch, Salome Oeri, Tomasz Kujawski, Zsolt Szucs-Farkas, Rainer Zbinden, Dominik P. Guensch, Kady Fischer

**Affiliations:** ^1^ Cardiology, Hospital Centre of Biel, Biel, Switzerland; ^2^ Department of Anaesthesiology and Pain Medicine, Inselspital, Bern University Hospital, University of Bern, Bern, Switzerland; ^3^ Radiology, Hospital Centre of Biel, Biel, Switzerland; ^4^ Department of Diagnostic, Interventional and Paediatric Radiology, Inselspital, Bern University Hospital, University of Bern, Bern, Switzerland

**Keywords:** quantitative perfusion, myocardial blood flow, fibrosis, stress-CMR, extracellular volume

## Abstract

**Introduction:**

Indications for stress-cardiovascular magnetic resonance imaging (CMR) to assess myocardial ischemia and viability are growing. First pass perfusion and late gadolinium enhancement (LGE) have limited value in balanced ischemia and diffuse fibrosis. Quantitative perfusion (QP) to assess absolute pixelwise myocardial blood flow (MBF) and extracellular volume (ECV) as a measure of diffuse fibrosis can overcome these limitations. We investigated the use of post-processing techniques for quantifying both pixelwise MBF and diffuse fibrosis in patients with clinically indicated CMR stress exams. We then assessed if focal and diffuse myocardial fibrosis and other features quantified during the CMR exam explain individual MBF findings.

**Methods:**

This prospective observational study enrolled 125 patients undergoing a clinically indicated stress-CMR scan. In addition to the clinical report, MBF during regadenoson-stress was quantified using a post-processing QP method and T1 maps were used to calculate ECV. Factors that were associated with poor MBF were investigated.

**Results:**

Of the 109 patients included (66 ± 11 years, 32% female), global and regional perfusion was quantified by QP analysis in both the presence and absence of visual first pass perfusion deficits. Similarly, ECV analysis identified diffuse fibrosis in myocardium beyond segments with LGE. Multivariable analysis showed both LGE (*β* = −0.191, *p* = 0.001) and ECV (*β* = −0.011, *p* < 0.001) were independent predictors of reduced MBF. In patients without clinically defined first pass perfusion deficits, the microvascular risk-factors of age and wall thickness further contributed to poor MBF (*p* < 0.001).

**Discussion:**

Quantitative analysis of MBF and diffuse fibrosis detected regional tissue abnormalities not identified by traditional visual assessment. Multi-parametric quantitative analysis may refine the work-up of the etiology of myocardial ischemia in patients referred for clinical CMR stress testing in the future and provide a deeper insight into ischemic heart disease.

## Introduction

As ischemic heart disease remains one of the most common and fastest growing causes of mortality, the number of stress examinations to investigate inducible ischemia are rapidly expanding and are being performed in imaging centers outside of university hospitals. Cardiovascular magnetic resonance imaging (CMR) is considered one of the most comprehensive diagnostic imaging modalities and the advancement of parametric tissue characterization techniques allows for a deeper investigation into diffuse myocardial dysfunction.

A typical clinical CMR exam for ischemic testing relies mostly on a combination of qualitatively assessing myocardial perfusion under stress or a vasodilating stimulus and imaging of permanent myocardial injury or viability of the tissue (1). A comprehensive understanding of any underlying perfusion and tissue abnormalities can better risk stratify a patient with suspected coronary artery disease as both features have established incremental prognostic value to eachother (2). Tissue viability is commonly assessed by late gadolinium enhancement (LGE). However, LGE is reliant on regional variability comparing enhanced myocardium to supposedly normal tissue and is ideal for investigating replacement scar associated with infarct, but it can consequently overlook diffuse fibrosis. Calculation of extracellular volume (ECV) through parametric T1 maps can overcome this limitation to non-invasively depict the fibrotic burden per voxel (3). Similarly, the routine clinical analysis of myocardial perfusion commonly includes qualitative first pass perfusion (FPP) analysis, where delays in the arrival of contrast agent to the myocardium are visually assessed and classified as a perfusion deficit. FPP also performs poorly in balanced ischemia in three-vessel disease or microvascular dysfunction because of diffusedly decreased contrast agent uptake in all or most segments (4), and detection of perfusion deficits relies heavily on the experience of the examiner (5). As a result, there is increased interest into development of quantitative perfusion (QP) techniques. With a fully quantitative analysis, absolute myocardial blood flow (MBF) is assessed for each pixel and expressed in milliliters per gram of myocardial tissue per minute (ml/g/min) (6) thereby it is not reliant on visual appreciation of regional differences. In comparison to T1 and ECV mapping, QP is not yet as established and its implementation is limited. There are a variety of QP techniques available, with many publications using novel sequences with inline MBF analysis. However, access to these specialized sequences may not be as widely available outside of tertiary research centers. An alternative to increase access to QP could be to apply post-processing software techniques that quantify MBF from product first pass perfusion sequences (7–10).

Thus, we investigated the use of post-processing techniques for quantifying pixelwise MBF in comparison to standard clinical visual analysis of visual FPP in patients undergoing clinically indicated CMR stress testing. Moreover, to shed light on potential discrepancies in regional and global hypoperfusion between the two perfusion analysis techniques we investigated if patient characteristics and quantification of other myocardial features acquired within the same multi-parametric CMR could explain MBF findings acquired by QP post-processing analysis. In particular, the primary aim was to investigate if visually assessed focal scar and quantified diffuse fibrosis have an independent impact on QP-derived MBF under regadenoson stress.

## Methods

### Study design & patient enrolment

The study was approved by the ethics board of the Canton of Bern, Switzerland (2021_00139) and complies with the Declaration of Helsinki. All subjects had given their written informed consent before enrolment into this study. This was a prospective observational study that enrolled 125 patients at the Hospital Centre of Biel, Switzerland who were referred for a clinically indicated stress-CMR to investigate inducible ischemia (May 2021–May 2022). Exclusion criteria were caffeine consumption in the last 12 h, <18 years of age, inability to give consent, or patients deemed unfit for an extended protocol by the imaging physician.

### CMR exam

**Figure 1:**
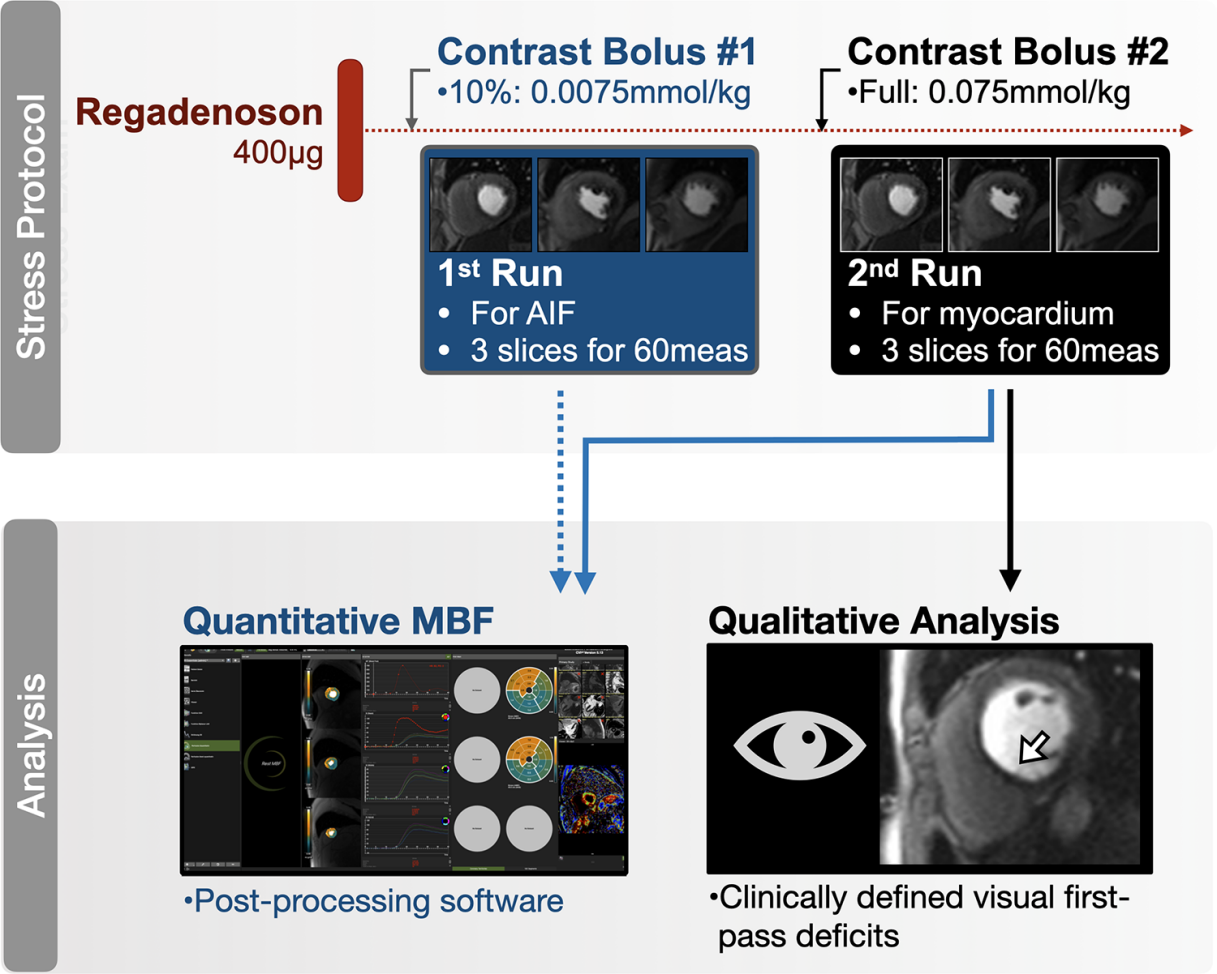
Quantitative perfusion protocol. Standard perfusion sequences were run twice while under regadenoson stress; first with 10% of the contrast agent to acquire the images used for the arterial input function (AIF), then repeated using the full contrast agent bolus for the main myocardial perfusion sequence. Both series were used for the quantitative (blue) myocardial blood flow (MBF) analysis (Supplementary Video 1), while only the second series was used for clinical (black) analysis of first-pass perfusion deficits.

The CMR studies were performed using a clinical 1.5 T MRI (Magnetom Sola, Siemens Healthineers). Patients underwent cine imaging with a short axis stack and three long-axis views for functional analysis. Stress was induced with intravenous administration of regadenoson (fixed dose of 400 μg), and perfusion at stress was imaged using a dual bolus protocol (10, 11) (Figure 1). FPP images were acquired with a standard product gradient echo-based pulse sequence in three short-axis slices, initially using a 10% bolus of contrast agent required for QP (0.0075 mmol/kg, Gadovist, Bayer) for determining the arterial input function (AIF). The sequence was then repeated with the full contrast agent dose (0.075 mmol/kg). After stress the remaining contrast agent was given to reach a total dose of 0.15 mmol/kg, and LGE images were acquired for the assessment of focal scar eight minutes after the third bolus. T1 maps in three short-axis slices were acquired prior to and after contrast agent injection for the calculation of ECV as a measure of diffuse fibrosis. Sequence details are provided in the Supplementary Methods.

### CMR image analysis

Images were analyzed by three independent groups of readers. First, a clinical analysis was performed by the local imaging team of senior physicians with CMR certification and >5 years of CMR experience. The clinical report included volumetric function, septal wall thickness and a visual assessment of the presence of LGE and qualitative FPP deficits. The presence or absence of the latter two parameters were reported for each myocardial segment [American Heart Association (AHA) 16-segment model] and classified by distribution pattern. A semiquantitative segment score was calculated for both measures based on the sum of segments with either LGE enhancement or visual FPP deficits. A patient was reported to have clinically relevant inducible ischemia by the presence of two adjacent segments with visual FPP deficits and have clinically relevant LGE if any myocardial enhancement was detected.

For quantitative analysis performed for research purposes, images were then recoded. Then QP and T1 maps were each analyzed by blinded readers, who did not take part in the first reading session. QP and T1 maps were both analyzed using CVI^42^ (Circle Cardiovascular Imaging, version 5.13). For the quantitative perfusion contours were added automatically and adjusted by the reader if required. Hyperemic myocardial blood flow (MBF) per voxel was then quantified by the software and reported as numerical result per AHA segment and for a global MBF (8, 10). Pixel-wise MBF maps were also generated and used for the correction of contours (Supplementary Video 1). Ten datasets were randomly selected and reanalyzed for a blinded intra- and interobserver assessment each. Pre- and post-contrast T1 maps were analyzed for myocardial and blood pool signal and ECV was calculated and reported as percent (12). For categorical comparisons ECV was dichotomized by a cutoff of 30%.

### Statistical analysis

Patients were included in the final analysis if QP, ECV and LGE were all successfully acquired. For the primary endpoint, the relationship between other CMR findings and patient characteristics to global and segmental MBF were assessed with a univariable linear regression. Continuous variables were utilized for MRI measurements such as segment score for LGE and visual FPP, or % for ECV. Significant variables (*p* < 0.05) were then forwarded into a multivariable model. For secondary analysis investigating categorical comparisons, global and segmental MBF from QP analysis were grouped based on the presence of LGE and ECV ≥ 30% and compared with a multivariate ANOVA and Tukey's *post-hoc* tests accounting for multiple comparisons. Similar models were used to compare the visual and quantitative techniques. Quantified MBF (ml/g/min) was compared between segments categorized by the presence and distribution of visual FPP deficits. The same analysis was performed for ECV(%) in the presence of LGE. For tests using segmental analysis, multiple measures per patient were accounted for in the statistical models. Intra- and interobserver reliability was calculated with an intra-class correlation (ICC) test for absolute agreement. Results were considered statistically significant at a two-tailed value *p *< 0.05. Statistical analyses were performed with GraphPad Prism version 9.0 (GraphPad Software) and *R* software (version 3.5.0, R Foundation for Statistical Computing).

## Results

### Patient characteristics and inclusion

From the 125 patients prospectively enrolled (Table 1), 109 were included in the final analysis (87%). In eight (6%) patients either the AIF pre-bolus sequence or T1 maps were not acquired, in five (4%) patients it was decided during the CMR to not give pharmacological stress, and in three (2%) patients, there was an error with the software and QP could not be calculated (Supplementary Figure S1). Patient characteristics are provided in Table 1, mean age was 66.5 ± 10.9 years, 34% were females. Hypertension (58%) and dyslipidemia (51%) were the most common comorbidities. Left ventricular ejection fraction was 60.1 ± 12.7%, cardiac output of 5.9 ± 2.2 L/min and a septal wall thickness of 10.8 ± 2.7 mm.

**Table 1:** Patient characteristics.

	Total (*n* = 109)
Age, years	66.5 ± 10.9
Sex (females)	34 (32%)
Body mass index, kg/m^2^	28 ± 5.2
Indication for stress-CMR	
Stable angina pectoris/ECG abnormalities	51 (47%)
Dyspnea/exercise intolerance of unclear origin	17 (16%)
Suspected ischemia secondary to other disease	15 (14%)
Follow-up of known CAD	13 (12%)
Echocardiography abnormalities	7 (6%)
Other	6 (5%)
Comorbidities	
Hypertension	63 (58%)
Dyslipidemia	56 (51%)
Diabetes mellitus	29 (27%)
Sleep apnea syndrome	16 (15%)
History of CAD	
*Myocardial infarction*	18 (17%)
*Coronary intervention*	31 (28%)
Atrial fibrillation	12 (11%)
Obesity	25 (23%)
Infiltrative diseases	0 (0%)
Medication	
Anti-platelets	36 (33%)
Dual anti-platelets	9 (8%)
Anti-coagulants	19 (17%)
ACE inhibitors	28 (26%)
Angiotensin II receptor blockers	36 (33%)
ARNI	2 (2%)
Calcium channel blockers	22 (20%)
Spironolactone	4 (4%)
SGLT-2 inhibitors	9 (8%)
Beta-blockers	44 (40%)
Diuretics	31 (28%)
Anti-lipid therapy	55 (50%)

Mean ± standard deviation, or frequency (percentage) are shown. ACE, angiotensin-converting-enzyme; ARNI, angiontensin receptor neprilysin inhibitor; CAD, coronary artery disease; ECG, electrocardiogram; SGLT-2, sodium-glucose cotransporter-2.

### Comparison of visual and quantitative analysis

**Figure 2:**
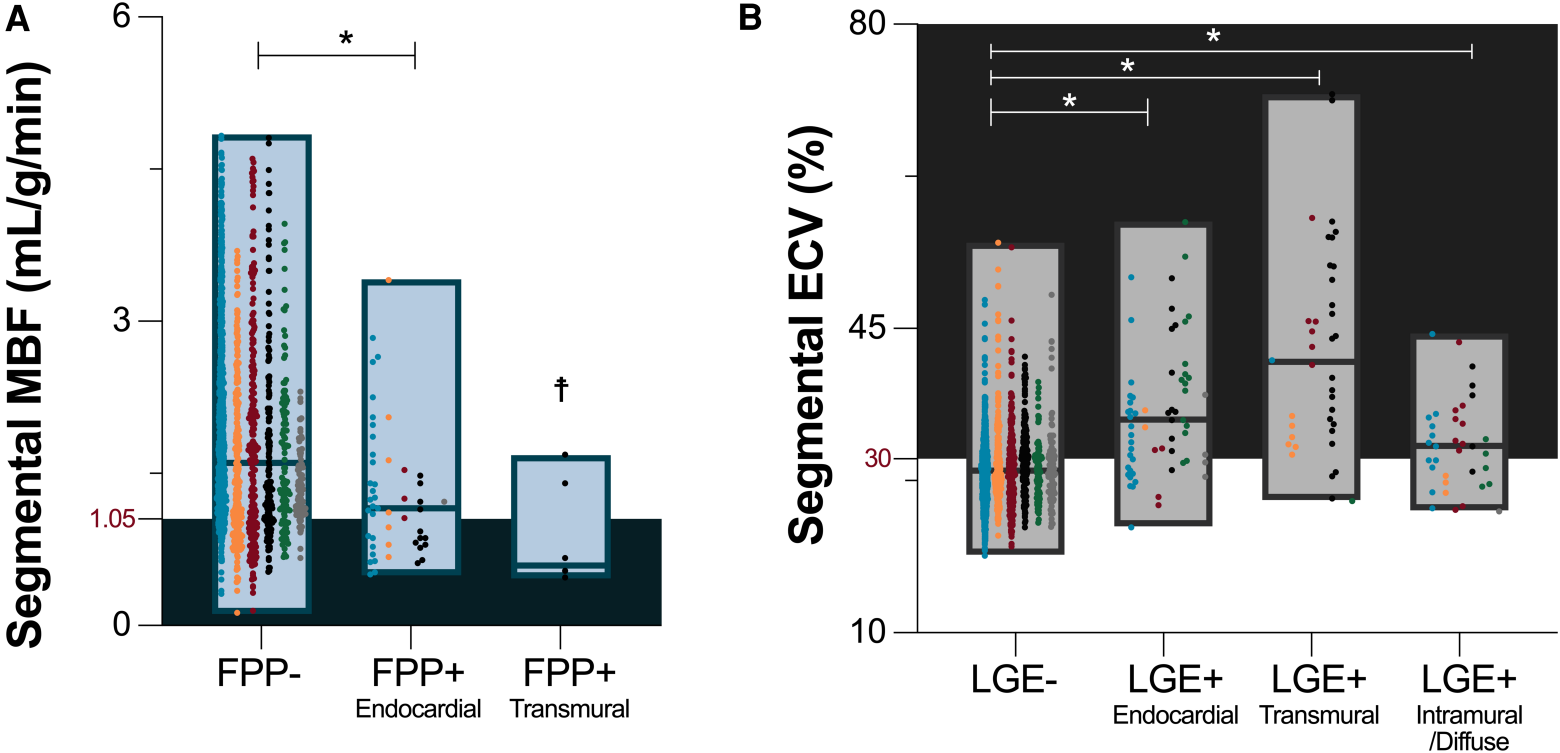
Visual versus quantitative analysis. (**A**) Median and range of myocardial blood flow (MBF) assessed by quantitative perfusion is depicted for segments with no categorized visual first pass perfusion (FPP) deficits, and in segments with an endocardial or transmural visual FPP deficit. As cut-offs are not yet defined for this technique, the shaded territory highlights MBF below the median calculated in segments with a visual FPP deficit (1.05 ml/g/min). (**B**) Median and range of quantitative extracellular volume (ECV) measurements are shown based on the absence of late gadolinium enhancement (LGE-), and by the presence of LGE(+) and distribution pattern. Shaded territory represents abnormal ECV, >30%. Data points are further colored by the primary indication for a stress CMR listed on the referral: unexplained angina pectoris and/or electrocardiogram abnormalities (blue), unexplained dyspnea and/or exercise intolerance (orange), suspected ischemia secondary to other cardiovascular diseases (red), follow up of known coronary artery disease (black), wall motion abnormalities in echocardiography exams (green), other (grey). *p < 0.05 in comparison to myocardial segments without visually classified tissue abnormalities (FPP-/LGE-). ☨Statistical analysis was not performed for FPP + transmural segments due to low sample size (*n* = 4).

All included patients presented with signs of a sufficient hemodynamic response during regadenoson, defined by an increase in heartrate of ≥10 bpm (18 ± 7 bpm) and/or a drop in systolic blood pressure of ≤−10 mmHg [−5.0 (−16-2.5)mmHg]. Of the 109 patients, 22 (20%) had a visually classified FPP deficit in at least one myocardial segment, and 10 patients (9%), were diagnosed with a clinically relevant inducible ischemia, defined by adjacent segments with FPP deficits. Regionally, the majority of segments with a visual FPP deficit were classified with endocardial hypoperfusion (*n* = 50), while the remainder had transmural hypoperfusion (*n* = 4). MBF was higher in segments without visual FPP deficit [1.6 (1.1–2.3 ml/g/min)], than those with a classified FPP deficit [1.2 (0.8–1.6)ml/g/min, *p* < 0.001]. Yet as shown in Figure 2, the range of MBF in visually classified normal segments was large with a clear overlap between segments with FPP deficits.

For the assessment of fibrosis, LGE was present in 41(38%) patients. In these patients presenting with LGE, enhancement was observed in a median of 3 [1–5] AHA segments (/16). Of the 132 total segments with LGE, 63 were classified with a sub-endocardial distribution, 40 as transmural and 29 as intramural/diffuse. Quantitative analysis of ECV as a pixelwise measurements of diffuse fibrosis was lower in segments without LGE than with LGE [28.6 (26.2–31.0)% vs. 34.7 (30.5–40.9)%, *p* < 0.001]. This trend was significant, independent if the LGE distribution was sub-endocardial, transmural or intramural/diffuse (Figure 2). Similar to MBF and visual FPP analysis, the range of ECV in segments without LGE was large, and a portion of these segments (36%) were above the cutoff of 30% indicating these patients had fibrosis that was only detected by quantitative measurements and not the traditional LGE technique.

### Factors impacting global myocardial blood flow

**Figure 3:**
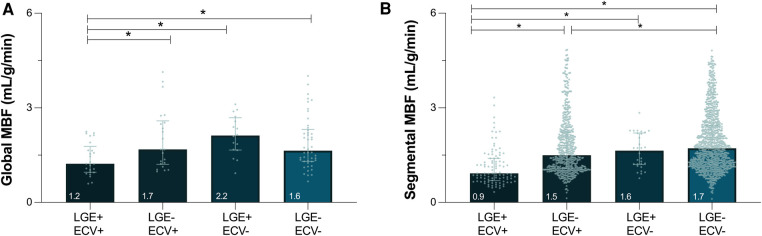
Myocardial blood flow in the presence of focal and diffuse fibrosis. Median [interquartile range] depict global (**A**) and segmental (**B**) myocardial blood flow (MBF) measured by quantitative perfusion, stratified by the presence of focal (LGE, late gadolinium enhancement) and/or diffuse fibrosis (ECV ≥ 30%, extracellular volume). *p < 0.05 between groups.

**Figure 4:**
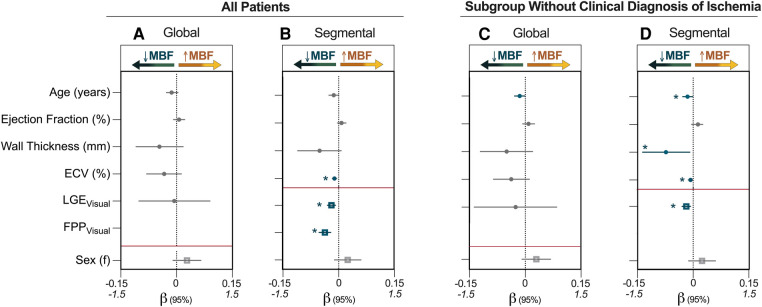
Multivariable analysis identifying factors associated with MBF. (**A**) and (**B**) represent the entire patient cohort (*n* = 109), while (**C**, **D**) are the subset of patients who were classified by visual analysis as not having inducible ischemia defined by two adjacent segments with a first-pass perfusion (FPP) deficit (*n* = 99). Regression coefficients (β) and 95% confidence intervals from multivariable analysis are shown for the independent association of variables to myocardial blood flow (MBF, ml/g/min). For global measurements, late gadolinium enhancement (LGE) is quantified using segment scores, which indicates the number of AHA segments (/16) that were determined in the clinical analysis to visually have enhancement whereas segmental analysis used just presence of enhancement in the individual segment for analysis. Continuous variables (circle) are plotted against the upper x-axis (*β*: −0.15 to 0.15), while categorical variables (square) are plotted against the lower x-axis (*β*: −1.5 to 1.5), and this is delineated by the red line. ECV, extracellular volume; f, females. *p < 0.05 in multivariable model (blue).

Median global MBF from the QP analysis was 1.6 (1.2–4.1)ml/g/min. Twenty-five (23%) patients presented positive for both LGE and ECV ≥30% averaged across the whole heart (LGE+/ECV+), 22 (20%) patients had elevated ECV but no LGE (LGE-/ECV+), 16 (15%) patients had LGE with ECV <30%, and 46 (42%) patients presented with no LGE or ECV elevation (Figure 3A). Global MBF during stress was lowest [1.2 (1.0–1.8) ml/g/min] in patients that presented with both a globally elevated ECV and LGE in comparison to all other categories (*p* < 0.05 vs. all). However, there was no difference between the other three categories, even if there was either LGE or ECV abnormalities [LGE-/ECV+: 1.7 (1.2–2.6), LGE+/ECV-: 2.2 (1.7–2.7), LGE-/ECV-: 1.6 (1.3–2.3) ml/g/min]. Univariable analysis investigating variables that impact MBF demonstrated that increased age, males, low ejection fraction and cardiac output, increased wall thickness, ECV(%) and number of segments with LGE all had significant associations (*p* < 0.05, all β- coefficients provided in Supplementary Table 1). In the multivariable analysis none of these factors were independently linked to global MBF (Figure 4).

### Factors impacting segmental myocardial blood flow

When investigating individual segments (*n* = 1,695), 6% had both LGE and ECV ≥ 30% (LGE+/ECV+), 35% of the segments had ECV ≥ 30% but no LGE (LGE-/ECV+), only 2% of segments showed LGE with ECV < 30%, and 57% patients presented with no LGE or ECV elevation. Similar to global analysis, segments presenting with both LGE and ECV ≥ 30% had a MBF of 0.9[0.6–1.4]ml/g/min, which was significantly lower than the three other categories (*p* < 0.05 vs. all). Moreover, even in the absence of LGE, segments with ECV ≥ 30% yielded lower MBF than segments without diffuse fibrosis [LGE-/ECV+: 1.5 (1.1–2.2)ml/g/min vs. LGE-/ECV-: 1.7 (1.2–2.4)ml/g/min, *p* = 0.041], whereas the few segments with LGE+/ECV- did not differ [1.6 (1.2–2.2)ml/g/min, *p* = 0.329].

**Figure 5:**
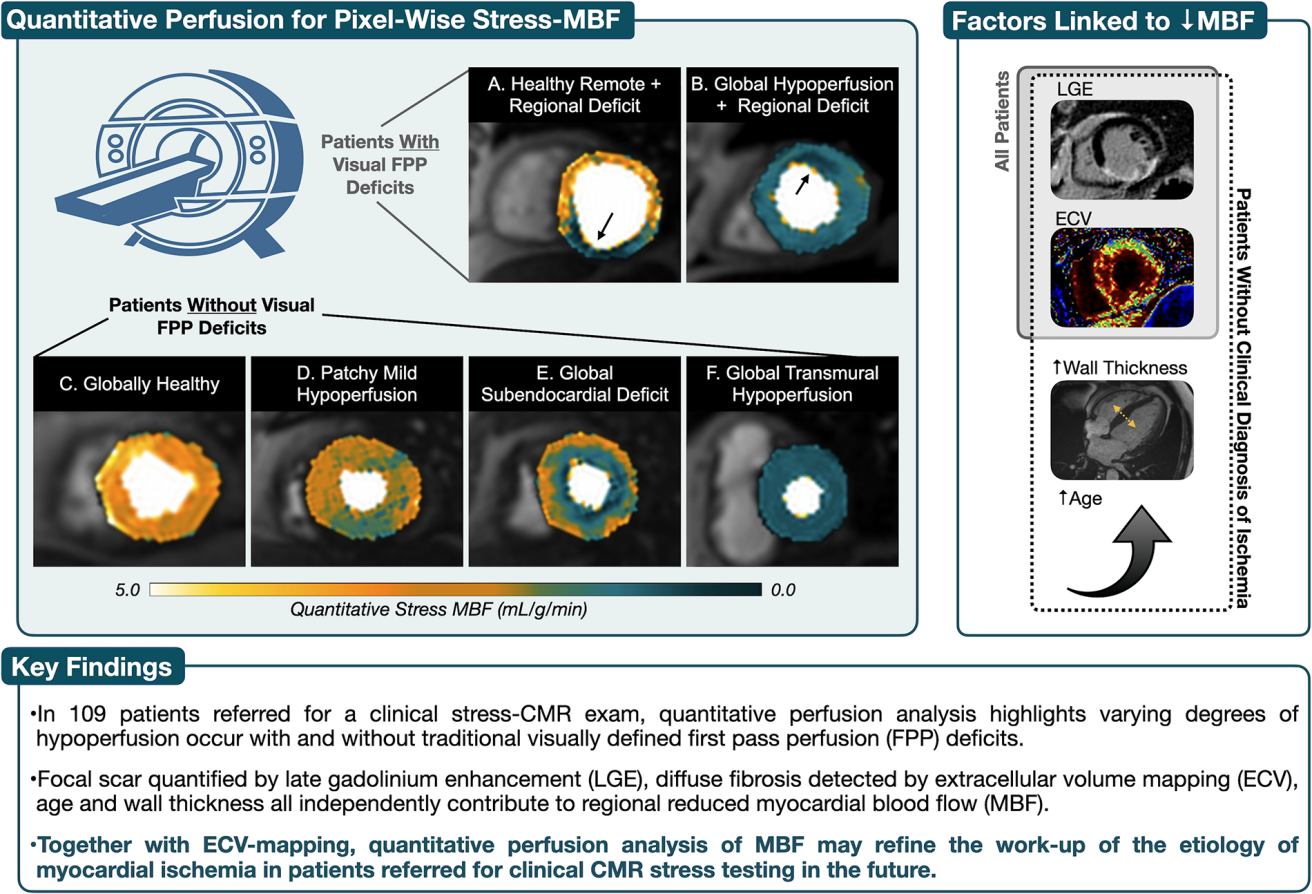
Take-home figure. Using the standard first pass perfusion (FPP) sequences, images were acquired under regadenoson vasodilation using a dual-bolus protocol and myocardial blood flow (MBF) was calculated using quantitative perfusion post-processing software. (**A**) and (**B**) both depict subendocardial perfusion deficits associated with coronary artery perfusion territories that were also detected by visual first pass perfusion (FPP) analysis. However quantitative perfusion maps help show that in A the remainder of the myocardium shows healthy MBF at stress, while patient B yields both a global reduction in MBF with a regional deficit of even lower MBF. Visual FPP deficits were not detected in patients (**C**–**F**), yet poor MBF can occur due to balanced and/or diffuse distribution of hypoperfusion. In these patients, the presence of focal (LGE, late gadolinium enhancement) and diffuse (ECV, extracellular volume) fibrosis along with increased wall thickness and age, all independently contribute to hypoperfusion.

Univariable segmental associations are provided in Supplementary Table 2. In the multivariable analysis, the presence of a visual perfusion deficit (*β* = −0.370, *p* < 0.001), LGE (*β* = −0.191, *p* = 0.001) and ECV (*β* = −0.011, *p* < 0.001) all independently explained the MBF accounting for multiple measurements per subject. Taking this one step further, univariable and multivariable analysis was again performed for the subset of patients (*n* = 99) that did not have clinical classification of inducible ischemia (two or more adjacent segments with an FPP deficit). In these patients, presence of LGE (*β* = −0.183, *p* = 0.002) and elevated ECV (*β* = −0.007, *p* = 0.035) still independently explained a lower segmental MBF, but in this subgroup increased age (*β* = −0.015, *p* = 0.040) and a larger wall thickness (*β* = −0.073, *p* = 0.030) were also independently associated with MBF (Figures 4, 5, Supplementary Table 3).

### Reader reliability of QP

**Figure 6:**
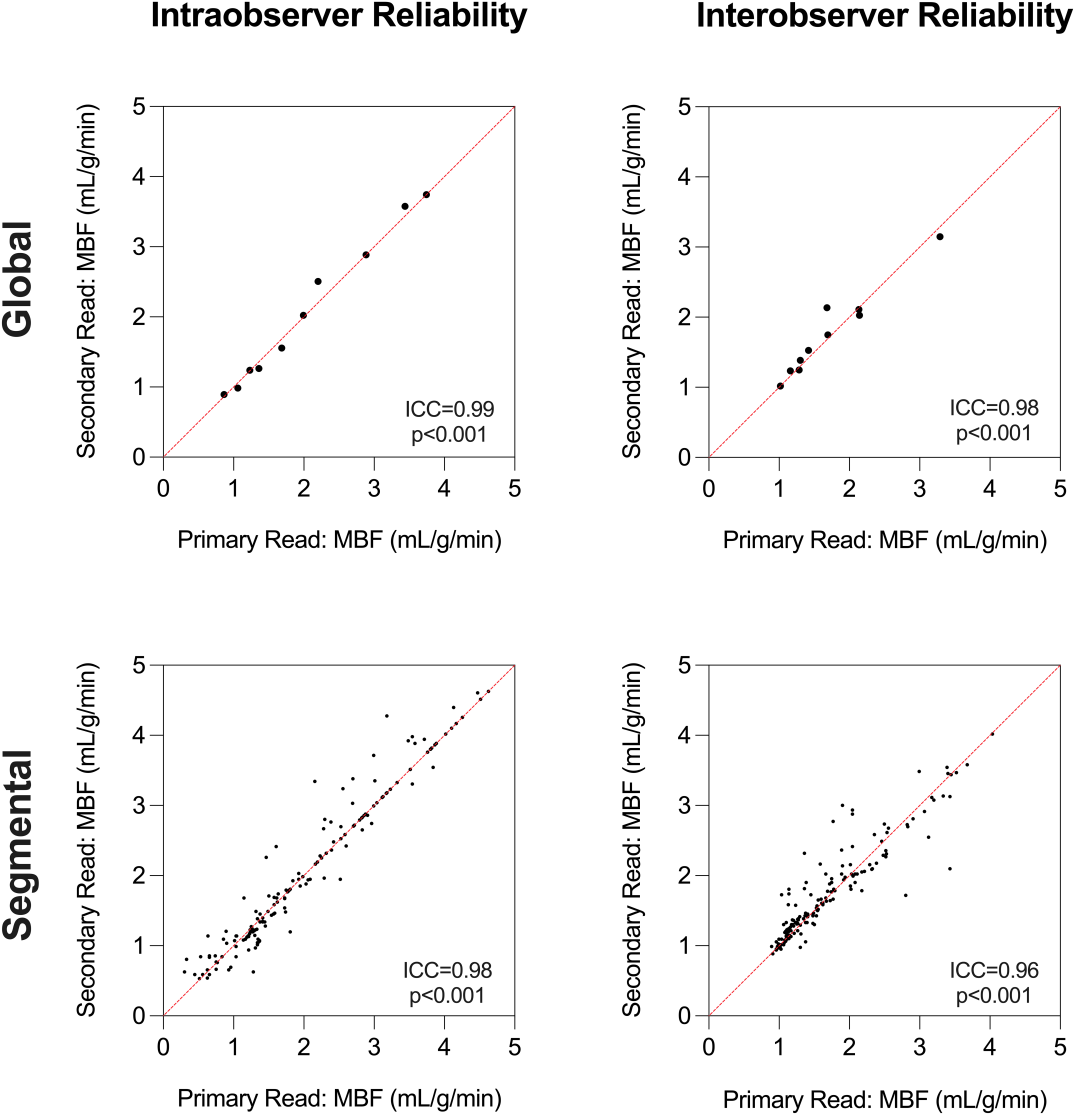
Reader reliability. Intraclass correlation coefficient (ICC) analysis demonstrates excellent intraobserver and interobserver reliability for global and segmental myocardial blood flow (MBF) measurements using quantitative perfusion analysis.

Reader reliability (Figure 6) for the QP measurements was excellent for intraobserver [ICC = 0.99 (0.98–0.99), *p* < 0.001] and interobserver [ICC = 0.98 (0.94–0.99), *p* < 0.001] global MBF quantification. Similar reliability was yielded for segmental MBF quantification [intraobserver: ICC = 0.98 (0.98–0.99), *p* < 0.001, interobserver: ICC = 0.96 (0.94–0.97), *p* < 0.001].

## Discussion

In a cohort of patients undergoing clinical stress CMR exams, analysis of pixel-wise MBF by post-processing quantitative perfusion (QP) software was feasible, reliable and was successfully performed in 97% of the acquired exams with pharmacological vasodilation. Hypoperfusion was identified in patients undergoing stress CMR, in both the presence and absence of visually assessed first pass perfusion (FPP) deficits. Even in patients who were not diagnosed with inducible ischemia by visual FPP analysis, both the presence of focal and diffuse fibrosis represented by the presence of LGE and ECV along with a thicker septal wall and patient age were independently associated with reduced regional MBF.

### Pixelwise quantitative analysis

In this study, both traditional qualitative and modern quantitative analysis techniques were applied to characterize the myocardial tissue features of perfusion and fibrosis. In the case of diffuse perfusion abnormalities and fibrosis patterns, abnormalities can remain undetected with the qualitative approaches because of the absence of healthy reference myocardium. This regional analysis technique can be compromised in the case of balanced impaired perfusion, arising from pathological processes including microvascular disease or 3-vessel CAD (4, 13). This could explain why only a portion of our patients received a clinical diagnosis of inducible ischemia from FPP analysis, despite suspicion of myocardial ischemia leading to the referral for a stress-CMR, and low MBF yielded from QP analysis. Similarly, others have shown visual FPP analysis underestimates the ischemic burden in multivessel disease, and quantitative perfusion was better at identifying the extent of two- and three-vessel disease CAD (13). QP isn't limited to highlighting balanced hypoperfusion but it can help clarify regional variation. For example in Figure 5, both panels A and B have a regional deficit visualized by FPP, yet in patient A the remainder of the myocardium has normal MBF with a small region of hypoperfusion, while in patient B, the entire myocardium is compromised with very low MBF in the anterior wall. These differences are patterns that cannot be distinguished by qualitative FPP analysis.

### Factors associated with decreased segmental MBF

Our results demonstrate the importance of segmental analysis. Averaging the entire myocardium into a single global MBF can lead to false negative results, if a regional perfusion deficit or a focal elevated ECV is compensated for or cancelled out by an otherwise healthy myocardium, resulting in a normal global value. Similarly, it was reported in hypertrophic cardiomyopathy patients that comparison of global MBF did not differ between patients with and without visual perfusion deficits, but investigation of individual segments revealed significant insights into regional decrease in perfusion (14). Thus, segmental analysis is beneficial to verify hypoperfusion, and to co-localize associated myocardial features (15–17).

In this cohort, as expected, the presence of a visual perfusion deficit and LGE were both associated with a co-localized segmental reduction in MBF. Hypoperfusion and tissue-injury have a mutually antagonistic relationship with each other. While poor blood flow can lead to myocardial ischemia and development of myocardial edema and fibrosis, structural damage to the tissue itself also impedes myocardial blood flow which subsequently can lead to further tissue injury (18). LGE excels at viability analysis and depicting replacement scar associated with myocardial infarction. Infarct scar is typically brought about by ischemic insults, in which myocytes are lost through necrosis and replaced by fibrotic scar. On the other hand, diffuse fibrosis often results as a reactive process of increased collagen deposition among myocytes, with typically less myocyte degradation (19). Consequently, LGE has lower accuracy in identifying these diffuse alterations in the myocardium (20). Diffuse fibrosis can impact the microvasculature and perfusion reserve through multiple mechanisms including stiffening of the ventricles leading to increased filling and intramural pressures, an increase in intercellular space and consequent change in microvascular architecture (21–23). Especially in the absence of infarct and obstructive macrovascular disease diffuse fibrosis can play a significant role in the microvascular dysfunction and microischemia (24, 25).

**Figure 7:**
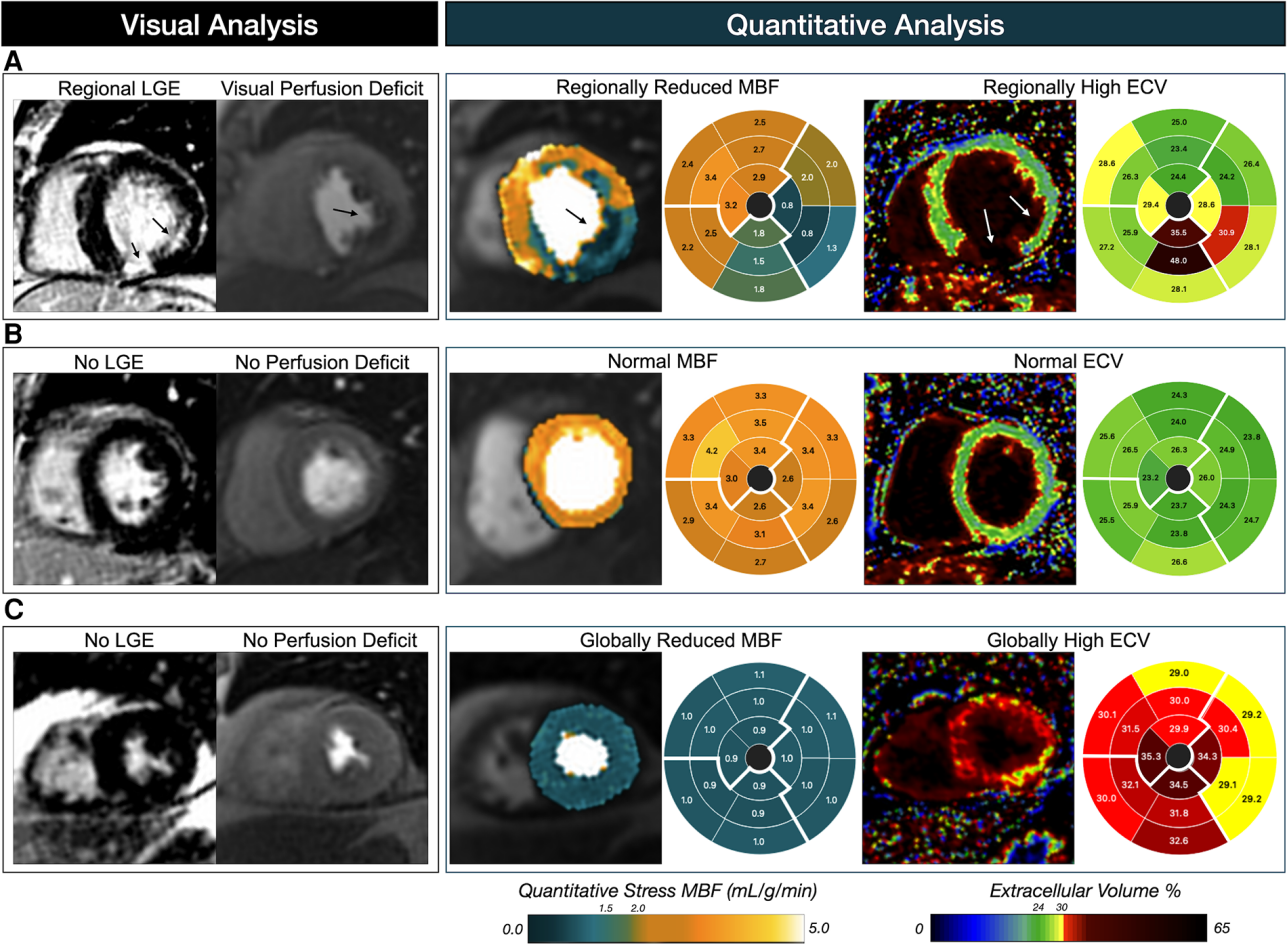
Patient examples of visual versus quantitative analysis. Patient (**A**) presents a classical CMR pattern associated with suspected coronary artery disease, in which a first-pass perfusion deficit and late gadolinium enhancement (LGE) are visualized in the infero-lateral wall depicting circumflex territory. Matching findings are observed with the pixel-wise myocardial blood flow (MBF) measurements from quantitative perfusion, and parametric extracellular volume (ECV) maps. Quantitative analysis of patient (**B**) is in agreement with the visual assessment with both normal MBF and ECV measurements across the myocardium. However, patient (**C**) represents the typical mismatch from visual assessment in which a homogenous low MBF is observed despite no visual perfusion deficits, and similarly ECV is elevated across the heart indicating a global diffuse fibrosis.

The fact the perfusion is attenuated in the presence of fibrosis is well established (26), but the difficulty had been that it was not manageable to routinely visualize and include measures of diffuse hypoperfusion and fibrosis into clinical reporting. The new aspect of this investigation is that we demonstrate that through post-processing methods, the hypoperfusion observed with QP and not FPP is influenced independently by both the traditional clinical LGE analysis and by the quantification of diffuse fibrosis. While ECV can depict the infarct core highlighted by LGE as seen in Figure 7, the parametric nature of this marker allows it to quantify additional myocardial abnormalities beyond the classical infarct-related scar (27). It should be noted, that ECV is not specific to myocardial fibrosis, but can also be seen with other myocardial injury as T1 and ECV maps can also depict edema and hemorrhage (3, 28–30).

We also observed in the sub-analysis that other factors beyond replacement and diffuse forms of myocardial fibrosis impact MBF. Increased age and larger wall thickness, both considered risk-factors for microvascular dysfunction, were also independently associated with reduced MBF. Measures of fibrosis along with increased wall thickness has already been linked to poor vascular function in specific pathological settings as in hypertrophic cardiomyopathy, Anderson-Fabry disease and HFpEF (31–33). Consequently, especially in patients who do not present with the typical ischemia pattern detected by FPP, i.e., subendocardial hypoperfusion localized to the center of one or two perfusion territories, hypoperfusion should not be discounted if these other imaging features associated with microvascular disease and global ischemia are present.

### Moving towards quantitative clinical analysis

QP has been primarily reserved to research settings, but multiple directions are currently developing techniques to translate QP to clinical routine such as through generation of in-line automated perfusion maps with respiratory motion correction, or through off-line software (4, 9, 34). Used for a clinical purpose, QP would potentially offer a more objective evaluation of ischemia and hypoperfusion.

This study uses a dual-bolus technique instead of the more validated dual-pulse single-bolus approach, where both the AIF and myocardial stress are acquired within the same acquisition. The main disadvantages of the applied dual-bolus technique is that it can be prone to T2* effects, it requires longer imaging time under stress as the sequence has to be repeated individually for the AIF and myocardial stress, and inline maps are not provided on the scanner (4, 35). However, a key advantage is that our approach does not require specialized sequences that may not be widely available outside of university hospitals. Rather we used the standard product sequence already implemented into our clinical routine and we were able to quantify MBF post-hoc with software without interfering with the traditional FPP images, which many clinicians still currently rely on for clinical decision making. For the current time, this technique presents a bridging option for imaging sites at secondary hospital or diagnostic centers which do not have access to developing sequences.

This combination of a dual-bolus protocol and post-processing software we have applied in our analysis was recently validated to fractional flow reserve measurements by Zhao et al. (10). Nevertheless, further comparisons between the approaches are needed in the future, especially for detection of microvascular dysfunction. So far in research settings, optimal cut-offs ranging from 1.2–2.6 ml/g/min have been reported to identify and risk stratify CAD patients, yet these come from different approaches and pharmacological agents resulting in a wide range of normal values (4, 8, 10, 34, 36, 37). In our cohort, global median MBF was 1.6 ml/g/min with a lower and upper interquartile range of 1.2–4.1 ml/g/min respectively. Regional median MBF dropped to about 1.2 ml/g/min in segments with a FPP deficit and/or LGE+/ECV + segments, while a median of 2.2 ml/g/min was observed in segments with no fibrosis. These medians are in the lower range of reported MBF, especially in comparison to reports using adenosine (4, 10). While there is also variation in MBF among studies using regadenoson (8, 37, 38), independent studies have reported MBF similar to ours while using regadenoson stress; for example a mean stress-MBF of approximately 0.9 ml/g/min was reported in ischemic territory while MBF remote territory in CAD patients and in non-CAD patients was approximately 2.3–2.4 ml/g/min (8). Regardless, normal values and relevant cut-offs for QP need to be clarified for each technique, consequently in our analysis we did not stratify the MBF findings by a cut-off. Moreover, this approach can be combined with rest perfusion to quantify the myocardial perfusion reserve, which has been reported to be an independent prognostic marker from stress MBF when using adenosine (34). However, we did not perform rest perfusion in our study as it is not incorporated into the local stress imaging clinical protocol. This could be investigated by integrating a rest perfusion scan before stress perfusion in further studies in which scan time can be extended to include rest perfusion. Importantly, quantitative rest perfusion after a stress perfusion using regadenoson, which was used in our study, will need further validation due to its much-longer half-life than adenosine (39).

### Limitations

The used post-processing technique quantified a single transmural value per segment, and did not quantify regions of interests, sub-endocardial values or transmural gradients, which may provide more differentiated picture of the hypoperfusion (36, 41). Similarly with ECV, small regions of fibrosis within a myocardial segment can be overlooked by numerical-only analysis if the remainder of the voxels in the segment are overall normal. This could explain segments that have LGE enhancement but the averaged ECV(%) is <30%. Since both QP and ECV postprocessing methods output numerical findings and visual maps, a combination of quantitative and qualitative assessment of parametric maps may improve classification further. As this study did not collect outcome measures, it is also unclear if the MBF results are related to cardiovascular events. Nevertheless, early reports already demonstrate QP has prognostic value for major adverse cardiovascular events (34, 42). We did not acquire invasive measures of CAD, but this protocol has been externally validated to macrovascular disease measurements (10). This comparison between imaging and invasive measures will need to be investigated further, as the goal of our study was rather to analyze the overall prevalence of hypoperfusion in a patient cohort undergoing diagnostic stress imaging in a non-tertiary institute and to investigate the impact of colocalized myocardial features on MBF.

### Clinical relevance

We observed in patients referred for a clinical stress-CMR exam to test for inducible ischaemia, that post-hoc quantitative analysis detected hypoperfusion and diffuse fibrosis in myocardium beyond territories categorized as abnormal by traditional visual evaluation. Incorporating quantitative analysis into clinical work may help explain the symptoms that led to a referral for ischemic stress testing in the first place. This could be especially useful to patients who present with signs of ischemia without the traditional patterns of sub-endocardial or transmural FPP deficits and LGE. This includes investigation of pathologies such as microvascular disease, myocardial infarction with non-obstructive coronary arteries, hypertrophy among others. Use of traditional stress FPP has been shown to be a cost-effective measure by helping derive a suitable treatment plan (42, 43), and future studies can investigate if this remains true for QP analysis. Finally, the CMR studies were performed in a smaller regional hospital, demonstrating that upon further validation post-processing software and dual-bolus techniques allows imaging sites without access to specialized sequences to perform quantitative analysis, potentially facilitating future access to QP to smaller sites.

## Conclusion

Quantitative perfusion image acquisition is feasible in a clinical setting and post-processing techniques can quantify pixel-wise myocardial blood flow using product perfusion sequences. Applying QP techniques, it was determined that MBF during pharmacological vasodilation was reduced in some patients independent of conventional visual FPP classification. Moreover, it was shown that ECV as a quantitative measure of diffuse fibrosis is independently associated with reduced MBF beyond focal scar on LGE images, while patient age and myocardial wall thickness are also independent contributors to hypoperfusion. Multi-parametric quantitative analysis may refine the work-up of the etiology of myocardial ischemia in patients referred for clinical CMR stress testing in the future and provide a deeper insight into ischemic heart disease.

## Data Availability

The raw data supporting the conclusions of this article will be made available by the authors, without undue reservation.

## Supplementary material

Supplementary Video S1 Quantitative perfusion analysis. Example of steps required for the post-processing. In this study only stress perfusion was acquired. Processing performed using a 2018 MacBook Pro with a 2.7 GHz Quad-Core Intel Core i7 processor.
