# Supplementary material for: Focal and diffuse myocardial fibrosis both contribute to regional hypoperfusion assessed by post-processing quantitative-perfusion MRI techniques
Source: Front Cardiovasc Med. 2023 Sep 19;10:1260156. doi: 10.3389/fcvm.2023.1260156 (PMC10546174; doi:10.3389/fcvm.2023.1260156)
Supplement: Supplementary file 1 [file Datasheet1.pdf]

SUPPLEMENTAL MATERIAL

Focal and diffuse myocardial fibrosis both contribute to regional hypoperfusion assessed by post-processing quantitative-perfusion MRI techniques

Table of Contents

Supplemental Methods: 2

Supplemental Figure 1: Consort figure....................3

Supplemental Table 1: Association to global quantitative myocardial blood flow in all patients 4

Supplemental Table 2: Association to segmental quantitative myocardial blood flow in all patients 5

Supplemental Table 3: Association to quantitative myocardial blood flow in patients with clinically assessed “no inducible ischemia”(n=99)....................6

Supplemental Methods:

Imaging Parameters

Images for functional analysis were obtained covering the ventricles with 8 to 14 short-axis (SAX) slices, and two to three long-axis views using a standard ECG-gated balanced steady-state free precession (bSSFP) cine sequence (echo-time 1.36ms, flip angle 65°, voxel size 1.9x1.9x6.0mm, bandwidth 930Hz/Px, 25 phases). Stress perfusion images were acquired using the product gradient echo based pulse sequence, and both arterial input function (AIF) and the main acquisition were acquired with 60 frames, this was increased up to 90 frames if left ventricular ejection fraction was estimated to be below 35% (2 proton density maps, echo time 1.26ms, flip angle 5°, voxel size 2.3x2.3x10.0mm, bandwidth: 651Hz/Px). T1 maps were imaged with a 5(3)3-modified Look-Locker sequence, (MOLLI: flip angle 35°, voxel size 1.6x1.6x8.0mm, bandwidth 1042Hz/Px). After a TI scout to determine inversion times for nulling of normal myocardium, LGE imaging was performed 8-12minutes post-contrast with slices orientated in a short-axis stack (8-12 slices) and two to three long-axis views (single shot, echo time 3.35ms, flip angle 26°, voxel size 1.3x1.3x8.0mm, bandwidth: 140Hz/Px).

Supplemental Figure 1: Consort figure

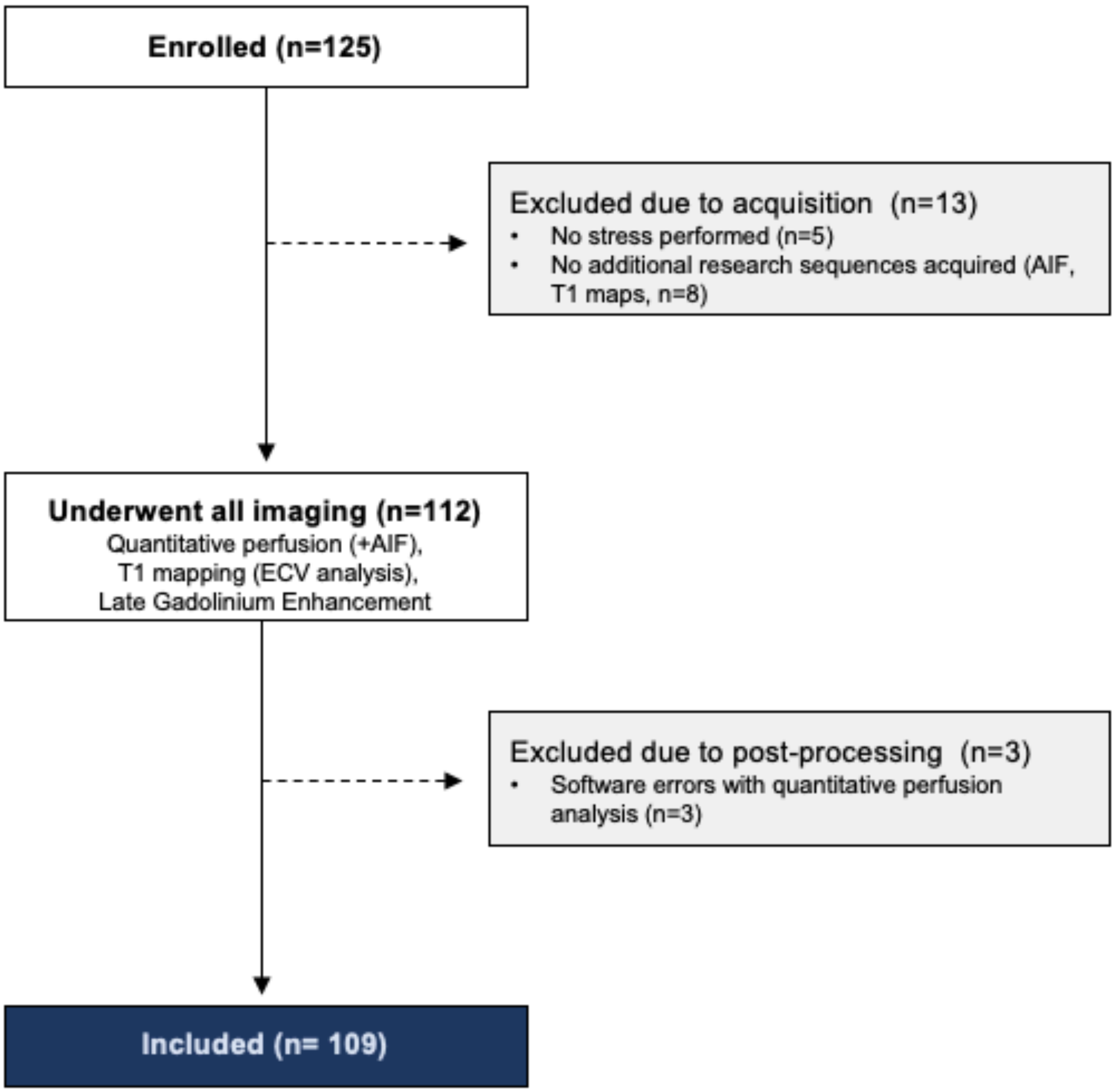

AIF: arterial input function sequence acquired prior to the standard perfusion sequence to allow for quantitative analysis. ECV: extracellular volume, calculated from pre-contrast and post-contrast T1 maps.

Supplemental Table 1: Association to global quantitative myocardial blood flow in all patients

	Univariable		Multivariable	
	**β (slope) (95%)**	**p**	**β (95%)**	**p**
**Medical History**				
Age (years)	-0.013 (-0.027, 0.010)	0.060	-0.013 (-0.027, 0.000)	0.056
Sex (females)	0.398 (0.075, 0.722)	0.016*	0.281 (-0.095, 0.657)	0.142
Diabetes	-0.194 (-0.540, 0.153)	0.270		
Hypertension	-0.148 (-0.455, 0.159)	0.340		
Previous Myocardial Infarction	-0.062 (-0.476 0.352)	0.767		
Previous Coronary Intervention	-0.202 (-0.541, 0.137)	0.239		
Obesity	0.241 (-0.127, 0.610)	0.197		
Dyslipidaemia	-0.229 (-0.534, 0.076)	0.139		
Body Mass Index (kg/m^2)	-0.012 (-0.041, 0.017)	0.418		
**CMR**				
Ejection Fraction (%)	0.016 (0.004, 0.028)	0.008*	0.007 (-0.008, 0.022)	0.329
Cardiac Output (L/min)	0.105 (0.037, 0.173)	0.003*		
Wall Thickness (mm)	-0.078 (-0.134, -0.022)	0.006*	-0.046 (-0.109, 0.018)	0.155
LV Mass index (g/m^2)	-0.003 (-0.0123 0.006)	0.508		
LGE Presence	-0.259 (-0.573, 0.054)	0.104		
LGE Segment Score	-0.104 (-0.178, -0.029)	0.007*	-0.006 (-0.101, 0.090)	0.908
ECV (%)	-0.051 (-0.092, -0.009)	0.018*	-0.033 (-0.080, 0.013)	0.155
Visual Perfusion Deficit Present	-0.037 (-0.570, 0-496)	0.892		
Visual Perfusion Segment Score	-0.052 (-0.171, 0.066)	0.383		

Regression coefficients (β) and 95% confidence intervals are shown for the association of variables to myocardial blood flow (mL/g/min) assessed by quantitative perfusion for global measurements in all patients (n=109). Wall thickness is determined by the interventricular septum in diastole, and perfusion and LGE segment scores represent the number of AHA segments (/16) that were determined visually in the clinical report to have perfusion deficits or enhancement. ECV: extracellular volume, LGE: late gadolinium enhancement, LV: left ventricular.

*p<0.05

Supplemental Table 2: Association to segmental quantitative myocardial blood flow in all patients

	Univariable		Multivariable	
	β (slope) (95%)	p	β (95%)	p
**Medical History**				
Age (years)	-	-	-0.013 (-0.026, 0.000)	0.060
Sex (females)	-	-	0.244 (-0.113, 0.602)	0.189
**CMR**				
Ejection Fraction (%)	-	-	0.009 (-0.003, 0.020)	0.147
Wall Thickness (mm)	-	-	-0.051 (-0.110, 0.007)	0.094
LGE Presence	-0.351 (-0.450, -0.252)	<0.001*	-0.191 (-0.301, -0.081)	0.001*
ECV (%)	-0.018 (-0.024, -0.012)	<0.001*	-0.011 (-0.017, -0.005)	<0.001*
Visual Perfusion Deficit	-0.525 (-0.674, -0.376)	<0.001*	-0.370 (-0.523, -0.213)	<0.001*

Regression coefficients (β) and 95% confidence intervals are shown for the association of variables to myocardial blood flow (mL/g/min) assessed by quantitative perfusion per AHA segment accounting for multiple segments per patient (n=109). Wall thickness is determined by the interventricular septum in diastole. ECV: extracellular volume, LGE: late gadolinium enhancement, LV: left ventricular
*p<0.05

Supplemental Table 3: Association to quantitative myocardial blood flow in patients with clinically assessed "no inducible ischemia"(n=99)

	Univariable		Multivariable	
	β (slope) (95%)	p	β (95%)	p
**GLOBAL**				
**Medical History**				
Age (years)	-0.015 (-0.029, -0.001)	0.047*	-0.015 (-0.029, -0.001)	0.041*
Sex (females)	0.400 (0.059, 0.742)	0.022*	0.296 (-0.086, 0.677)	0.127
Diabetes	-0.217 (-0.595, 0.161)	0.258		
Hypertension	-0.159 (-0.489, 0.171)	0.341		
Previous Myocardial Infarction	-0.006 (-0.481, 0.469)	0.980		
Previous Coronary Intervention	-0.202 (-0.591, 0.188)	0.307		
Obesity	0.253 (-0.141, 0.648)	0.206		
Dyslipidaemia	-0.218 (-0.546, 0.110)	0.190		
Body Mass Index (kg/m^2)	-0.013 (-0.043, 0.018)	0.400		
**CMR**				
Ejection Fraction (%)	0.020 (0.007, 0.033)	0.003*	0.009 (-0.007, 0.025)	0.270
Cardiac Output (L/min)	0.111 (0.039, 0.183)	0.003*		
Wall Thickness (mm)	-0.091 (-0.156, -0.025)	0.007*	-0.050 (-0.121, 0.020)	0.162
LV Mass index (g/m^2)	-0.003 (-0.014, 0.008)	0.564		
LGE Presence	-0.277 (-0.626, 0.072)	0.118		
LGE Segment Score	-0.132 (-0.225, -0.038)	0.006*	-0.026 (-0.138, 0.085)	0.638
ECV (%)	-0.056 (-0.102, -0.011)	0.016*	-0.038 (-0.086, 0.011)	0.126
**SEGMENTAL**				
Age (years)			-0.015 (-0.028, -0.001)	0.040*
Sex (females)			0.239 (-0.121, 0.600)	0.204
Ejection Fraction (%)			0.013 (-0.004, 0.026)	0.058
Wall Thickness (mm)	-0.096 (-0.157, -0.034)	0.003*	-0.073 (-0.134, -0.009)	0.030*
LGE Presence	-0.232 (-0.342, -0.125)	<0.001*	-0.183 (-0.296, -0.070)	0.002*
ECV (%)	-0.011 (-0.017, -0.005)	<0.001*	-0.007 (-0.013. -0.001)	0.035*

Regression coefficients (β) and 95% confidence intervals are shown for the association of variables to myocardial blood flow (mL/g/min) assessed by quantitative perfusion in the subset of **patients who did not have an inducible ischemia**, **determined by the clinical assessment of the presence of two adjacent segments with a visual first pass perfusion deficit (n=99).** ECV: extracellular volume, LGE: late gadolinium enhancement, LV: left ventricular

*p<0.05
